# Improved pointer in auditory alarms enhances response accuracy

**DOI:** 10.1016/j.bjao.2025.100379

**Published:** 2025-03-13

**Authors:** Michelle Shin, Ian Grant, Ramez Mikhail, Alexandra Lee, Tiffany-Chau Le, Alexandra Bruder, Judy Edworthy, Joshua Shive, Joseph J. Schlesinger

**Affiliations:** 1 Eastern Virginia Medical School, Norfolk, VA, USA; 2 University of Vermont Medical Center, Department of Anesthesiology, Burlington, VT, USA; 3 Vanderbilt University, Nashville, TN, USA; 4 Meharry Medical College, Nashville, TN, USA; 5 Ohio State University Medical Center, Columbus, OH, USA; 6 University of Plymouth, School of Psychology, Plymouth, UK; 7 Tennessee State University, Department of Psychology, Nashville, TN, USA; 8 Vanderbilt University Medical Center, Department of Anesthesiology, Nashville, TN, USA

**Keywords:** auditory icons, clinical alarms, IEC 60601-1-8, patient safety, pointers

## Abstract

**Background:**

Auditory alarms are crucial in clinical settings, alerting clinicians to events requiring immediate attention. However, multitasking can lead to missed alarms and disrupt patient care. Enhancing auditory alarms can improve patient safety and clinician satisfaction.

**Methods:**

In a controlled laboratory study, we recruited 26 clinicians (residents, fellows, advanced practice providers) and 19 non-clinicians (undergraduate students) to compare our previously validated alarm with an improved design. The improved alarm incorporates a ‘pointer’ (a short sound burst indicating acuity levels) enriched with harmony, intervallic change, roughness, and glissando to provide additional information to users. We measured response accuracy (correct alarm identification) and response time (seconds to respond).

**Results:**

A total of 26 clinicians and 19 non-clinicians were recruited and all participants met inclusion criteria for analysis. A mixed analysis of variance revealed a large main effect of the pointer on response accuracy (F(1,44)=9.11, *P*=0.004, η^2^_*p*_=0.17). Accuracy was higher for our improved pointer (M=0.90, 95% confidence interval [CI; 0.84–0.95]) than for our previous design (M=0.80, 95% CI [0.74–0.87]). Ascending alarms representing hypertension yielded a mean response accuracy of 0.89 (95% CI 0.84–0.94) and descending alarms representing hypotension yielded a mean response accuracy of 0.81 (95% CI 0.75–0.88). Low acuity ascending alarms resulted in slower response times compared with other combinations, where acuity of change was conveyed through intervallic difference of two-note harmonies.

**Conclusions:**

Improved pointers demonstrated statistically significant accuracy improvement for clinicians and non-clinicians without compromising response time—a design advantage that can influence the revision of the international alarm standard and improve patient safety.

## Background

Physiologic auditory alarms inform clinicians about critical events that may require immediate attention. Their purpose is to redirect attention to higher-acuity events,^1^ but excessive loudness or repetition can disrupt care and inflict undue stress onto both clinicians and their patients.^2^, ^3^, ^4^ Proposed solutions include voice alerts,^5^^,^^6^ spearcons,^7^ and auditory icons,^8^ the last of which were incorporated into the 2020 The International Electrotechnical Commission (IEC) update.^9^ All these alarm types can communicate the reason, direction, and priority of an alarm. Auditory icons effectively convey alarm type, direction, and priority but are difficult to design intuitively. Pointer sounds offer a practical alternative for prioritisation and direction.^10^

The IEC 60601-1-8, a subsection to its parent standard IEC 60601,^11^ governs alarms used in medical equipment to ensure basic safety and performance. Before its 2020 amendment, alarms were five-note ‘melodies’ which were difficult to learn and recognise^12^ in part because of technological limitations at the time. However, even within those constraints, the alarms were unnecessarily uniform from a human learning and recognition viewpoint.^7^^,^^12^

## Evolution of the pointer

The 2020 IEC update introduced auditory icons—real-world-like sounds representing their functions (i.e. ‘lub-dub’) representing a heartbeat for cardiovascular alarms.^13^ Auditory icons are diverse, intuitive, and easier to learn and localise than simple beeps.^14^, ^15^, ^16^ However, while icons aid problem identification (e.g. cardiovascular *vs* oxygen issues), they do not address acuity or direction. To address this, the updated alarms incorporate pointers—short sound bursts embedded within icons—indicating acuity levels (‘high’, ‘medium’, or ‘low’) through pulse counts: fast 5-pulses for high and slower 3-pulses for medium acuity.^9^ Here, we use the term ‘acuity’ to describe the extent or severity of the clinical condition, in line with its use in landmark initiatives such as the Surviving Sepsis Campaign.^17^ Initial studies^15^ showed similar performance with or without pointers; however, subsequent studies^16^ demonstrated that pointers enhance multitasking performance by reducing cognitive load and improving visual vigilance and speech intelligibility in noisy environments.

Aside from signifying high acuity, the 5-pulse did not convey additional information. Bruder and colleagues^16^ suggested that abstract pointers could communicate more if given greater harmonic complexity. A follow-up study by Bingham and colleagues^10^ explored this by testing medical alarms with two-note harmony *vs* single-note alarms with overtones. Participants responded faster to harmonic alarms, suggesting superiority of harmonic pointers in conveying acuity. A consonant-to-dissonant transition further improved pointer identification, particularly at higher frequencies. The study emphasised that incorporating harmony into alarm systems can enhance auditory cue recognition. These findings suggest that moving from simple melodic pointers to harmonically complex structures could improve alarm efficacy in healthcare, warranting further exploration of harmonic designs.

For these reasons, we have chosen to improve our existing design,^10^ focusing purely on the acoustic qualities of physiologic auditory alarms. In this study, we aim to compare the effect of our improved pointer to our previously validated auditory alarm^10^ on response accuracy and response time in clinician and non-clinician participants. We hypothesised that our improved pointer would improve both accuracy and response time in both clinicians and non-clinicians.

## Methods

### Study design

Here, we take the auditory pointer developed specifically for cardiovascular use previously utilised and tested in a laboratory-based validated paradigm in Bingham,^10^^,^^15^^,^^16^ and improve it with harmonic complexity, roughness (the harsh, raspy nature of narrow harmonic intervals best correlated with sensory dissonance), and glissando (auditory gliding that enables smooth flow from one note to another typically of different pitch) to create distorted sounds between the starting and ending notes to differentiate between low and high acuity.^10^ Because we have incrementally built upon a series of improvements, we have chosen to refer to our newest design as our ‘improved pointer’ throughout this manuscript. Our goal was to supplement the pointer with more information for the end user and make this auditory signal perceptually intuitive.^18^^,^^19^ Full psychoacoustic design details can be found, accompanied by sound files, in the Supplementary material.

**Fig 1:**
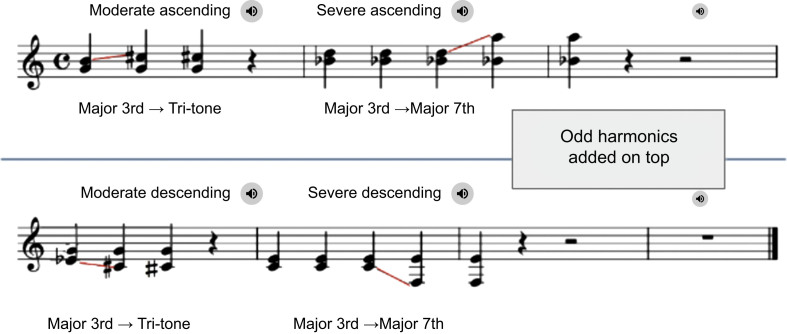
Musical representations of the improved alarm sounds displaying odd-number harmonics overlaying the base frequency note to create added roughness. Improved pointers include the addition of a glissando between the last two and the pre-penultimate tones in each case, ending with a fourth and fifth tone in the high acuity pointers (for example, from D to A for the severe ascending alarm sound). Severe sounds have a more syncopated (more emphasis on offbeats) feel compared with the moderate sounds.

We designate the four pointers tested in Bingham and colleagues^10^ as our ‘conventional’ pointers for comparison against our improved pointers. The tonal elements of the four pointers tested in Bingham and colleagues^10^ are as shown in Fig 1. Two-tone structure allows for additional differentiation between moderate and severe acuity by virtue of the severe acuity using a larger musical interval than moderate acuity. The musical intervals for those pointers begin with a major third (consonant) and go to a tritone (dissonant) for moderate conditions and from a major third to a major seventh (dissonant) for severe conditions. Blood pressure trends are indicated by the direction of movement of the two-tone sequence, so that upwards movement is indicated by a change of pitch upwards, and downwards movement is indicated by a change of pitch downwards. In testing, each of the pointers is preceded and followed by a short ‘lub-dub’ auditory icon, which does not differ for each of the four alarm conditions.

**Fig 2:**
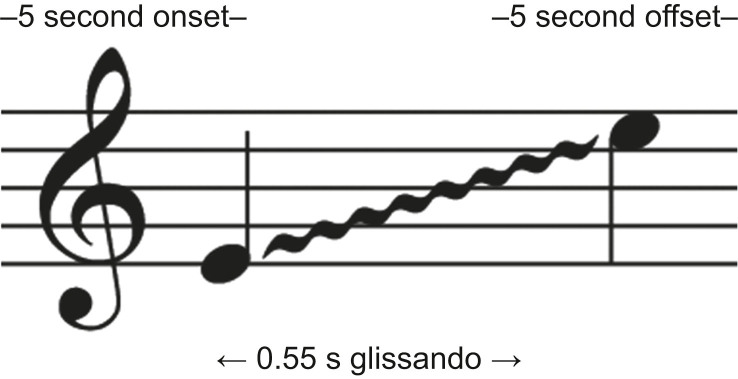
Illustration of a 0.55 s glissando sliding on a time scale. Glissando begins and ends at the highest note in the specific physiologic motif.

Though roughness cannot be notated in musical score, this was achieved by adding 3, 5, and 7 odd harmonics (an overtone that multiplies the fundamental sound frequency times an odd-numbered integer) to the final two tones. The other new element was a glissando, which is an auditory gliding enabling smooth flow from one note to another (typically of different pitch) to create distorted sounds between the starting and ending notes. Within the same metrical/time duration and rhythm as the current pointer stipulated in the international alarm standard, we wanted to maximise the expression of glissando without increasing the duration of the overall sound. Fifty-five millisecond glissandos (45 ms per note with a 5-ms attack and release) were added to each improved pointer (Fig 2). The low acuity alarms contained one glissando from the first to last note, effectively adding the semitone between the first and second tones of the signal. The high acuity glissando consists of six tones from the root to desired destination tone. The pulses of the low and high acuity pointers were presented every 2.24 s at a speed of approximately 26.8 beats min^−1^.

Our improved alarm was created using Audacity (Oak Park, MI, USA) and MAPLE Lab Auditory Exploration Suite for Teaching, Research, and Observation (MAESTRO, Trenton, NJ, https://maplelab.net/maestro/) software.

**Fig 3:**
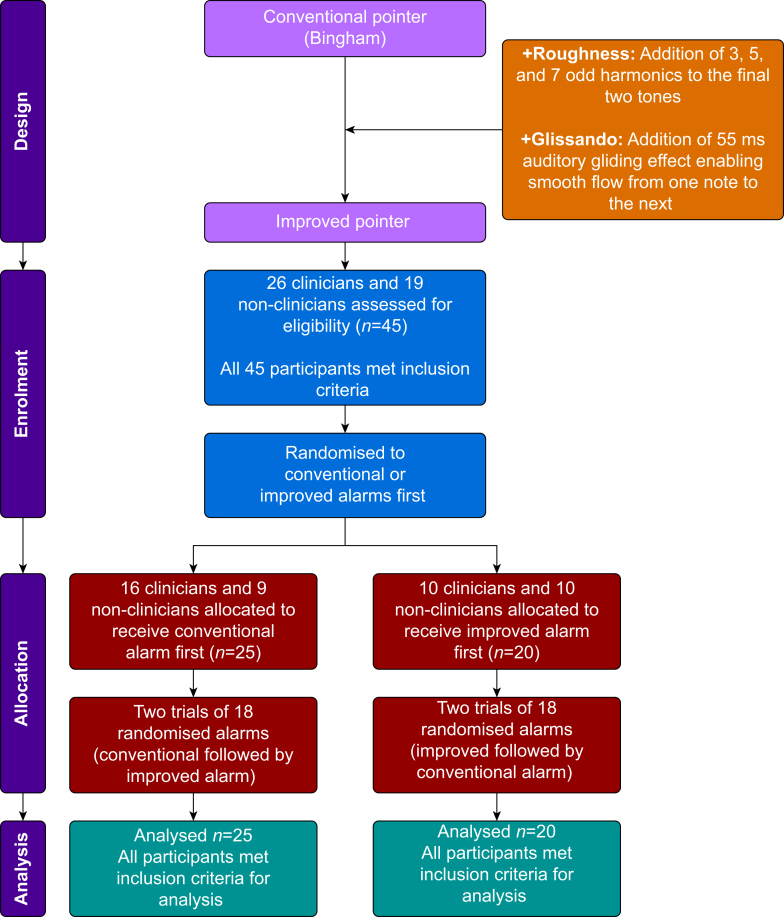
Flowchart illustrating the study overview, including the design, enrolment, allocation, and analysis phases. Participants were assessed for eligibility (*n*=45), randomised to receive either the conventional pointer alarm first (*n*=25) or the improved pointer alarm first (*n*=21), and underwent two trials of randomised alarms. Key features of the improved pointer alarm are noted. Final analysis included 16 participants from the conventional-first group and 22 participants from the improved-first group, with exclusions because of unmet inclusion criteria.

Clinician data came from a convenience sample of residents, fellows, and advanced practice providers recruited from the intensive care unit at Vanderbilt University Medical Center (VUMC). Non-clinician participants were recruited from Vanderbilt University's undergraduate population. A total of 26 clinicians and 19 non-clinicians were recruited (Table 1). This paper shows evolution of our design, and we have drawn from our previous studies with regards to the patient characteristics, methodological paradigm, and performance outcome metrics. Likewise, we have shown since 2018 and with this manuscript that we have been able to achieve statistical significance with our intended sample size. In a *post hoc* power analysis, we found that 45 participants provides ∼85% power to detect an effect size equal to that observed in the present study (i.e. F(1,44)=9.11). Inclusion criteria for the clinician participants included currently holding a license to practice as a medical professional at VUMC. Inclusion criteria for non-clinician participants included enrolment as an undergraduate student at Vanderbilt University. Exclusion criteria in both participant groups included active substance use and active hearing impairment. This research was approved by the Institutional Review Board at Vanderbilt University Medical Center (IRB Approval Number: IRB-230011) on 29 February 2023. All participants took part in this study voluntarily without being given compensation. Informed consent was obtained from each participant. This study adhered to the CONSORT (Consolidated Standards of Reporting Trials) guidelines for reporting randomised controlled trials. A completed CONSORT checklist is provided as Supplementary material. An overview of the study design is provided in Fig 3.

**Table 1:** Characteristics of each participant group, including age, sex, ethnicity, and baseline condition (hearing loss *vs* no hearing loss).

Characteristic	Clinicians	Non-clinicians	Total
Number of participants, *n*	26	19	45
Age (yr), mean (sd)	27 (3)	19 (1)	24 (5)
Sex, *n* (%)			
- Male	10 (38)	12 (63)	49
- Female	16 (62)	7 (37)	51
Ethnicity, *n* (%)			
- White	18 (70)	38 (76)	73 (73)
- Black or African American	4 (15)	8 (16)	18 (18)
- Other	4 (15)	4 (8)	9 (9)
Baseline condition, *n* (%)			
- Hearing loss	0	0	0
- No hearing loss	26 (100)	19 (100)	45 (100)

sd, standard deviation.

Participants were randomised to conventional or improved alarms first. After a short orientation including all four possible alarm conditions, patients identified randomly presented alarms while completing the Purdue Pegboard Test, manufactured by Lafayette Instruments (Lafayette, IN, USA), to simulate multitasking. Each participant completed two trials (conventional and improved alarms) with 18 randomised alarms per trial, separated by 15-s intervals. A total of 36 alarm sounds were presented, and each session, including orientation, lasted about 6.5 min. All participants performed the trial on a Dell XPS 15 laptop computer with Sennheiser HD 280 (Old Lyme, CT, USA) headphones calibrated on a multimeter to 7.0 mV, correlating to 70 dB at the loudest of the high acuity ascending pointer, ensuring detection well above the threshold of perception.

### Statistics

Primary outcomes of this study included response accuracy and response time in clinicians *vs* non-clinicians. Response accuracy was calculated as a fraction representing the number of correct responses out of the total responses. Response time was measured by tracking the time that elapsed between presentation of the alarm and the participant clicking a response on their laptop. Secondary outcomes included differences observed between alarm acuity, direction, or classification. After data collection, IBM SPSS Statistics 29 was used to perform two sets of data analysis using analysis of variance (anova). The first set of analyses examined the effects of pointer type and participant classification on response time and accuracy. The second set examined the effects of alarm characteristics (e.g. alarm direction, acuity, and alarm classification) and participant type on response time accuracy.

## Results

**Fig 4:**
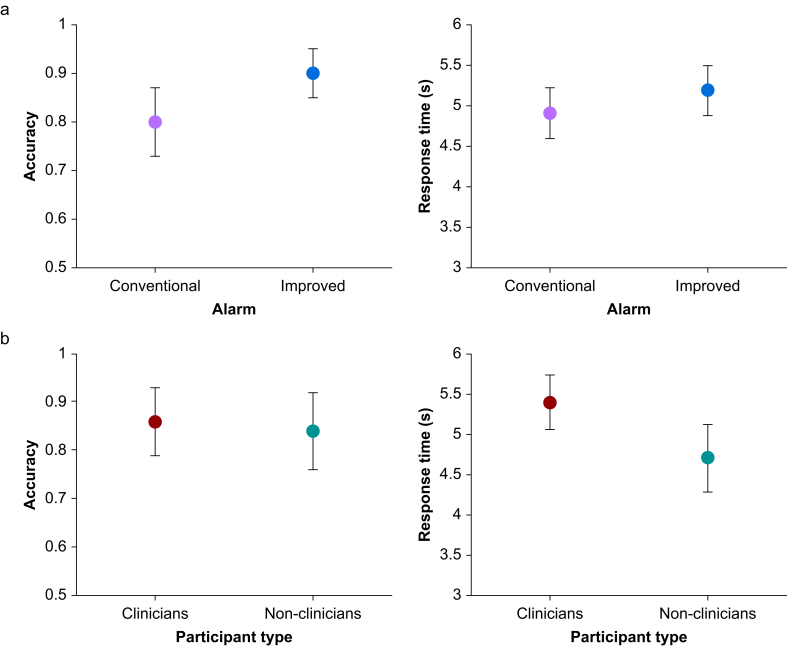
Accuracy and response time for (a) conventional and improved alarm pointer types and (b) clinicians and non-clinicians. The left figure of each pair shows the mean accuracy and 95% confidence interval for the mean for each group. The right figure of each pair shows the mean response time and 95% confidence interval of the mean for each group. Responses were more accurate with the improved pointer (upper-left figure) and faster for non-clinicians (lower-right figure).

There were no study dropouts and no missing data from any participant. The analyses presented in this section are based on the data from all 45 study participants. No participants were excluded from the analyses presented in this section. A mixed anova using response accuracy as the dependent variable, pointer type as a within-subjects factor, and participant classification (clinician, non-clinician) as a grouping variable revealed a large main effect of pointer type (*F*(1,44)=9.11, *P*=0.004, *η*^*2*^_*p*_=0.17). Response accuracy was higher for the improved pointers (mean accuracy=0.90, 95% confidence interval [CI 0.84–0.95]) than for the conventional pointers (mean accuracy=0.80, 95% CI 0.74–0.87), as shown in Fig 4. However, we did not find evidence for a main effect of participant classification or an interaction of pointer type and participant classification. The left-hand side of Fig 4 summarises these findings, with the upper figure showing the significant effect of pointer design on accuracy.

A similar analysis was carried out for the response time data. A mixed anova using response time as the dependent variable, pointer type as a within-subjects factor, and participant classification as a grouping variable showed a large main effect of participant classification (*F*(1,44)=6.44, *P*<0.05, *η*^*2*^_*p*_=0.13). Non-clinicians’ correct responses (mean response time=4.71 s, 95% CI 4.30–5.13 s) were faster than clinicians' correct responses (mean response time=5.40 s, 95% CI 5.05–5.74 s). No other main effect or interaction between factors was found. Our main findings from this analysis were that the improved pointer resulted in more accurate responses than the earlier pointer, and that non-clinicians responded more quickly than clinicians. The right-hand side of Fig 4 summarises these findings, with the lower figure showing the significant effect of pointer design on accuracy.

The effects of the pointer manipulations on performance were also analysed using the data from all 19 non-clinician participants and all 26 clinician participants. The mixed model anova with accuracy as the dependent variable, alarm direction, acuity, and alarm classification (improved *vs* conventional) as within-subjects factors and participant type as a between-subjects factor found large main effects of alarm direction (*F*(1,43)=13.08, *P*<0.001, *η*^*2*^_*p*_=0.23) and alarm classification (*F*(1,43)=10.14, *P*=0.003, *η*^*2*^_*p*_=0.19). Response accuracy was higher for ascending alarms (mean accuracy=0.89, 95% CI 0.84–0.94) than for descending alarms (mean accuracy=0.81, 95% CI 0.75–0.88). Furthermore, as in the previous accuracy analysis, response accuracy was higher for the modified pointers (mean accuracy=0.90, 95% CI 0.84–0.96) than for the conventional pointers (mean accuracy=0.81, 95% CI 0.74–0.87). We did not find sufficient evidence for any other main effects or interactions between factors.

**Fig 5:**
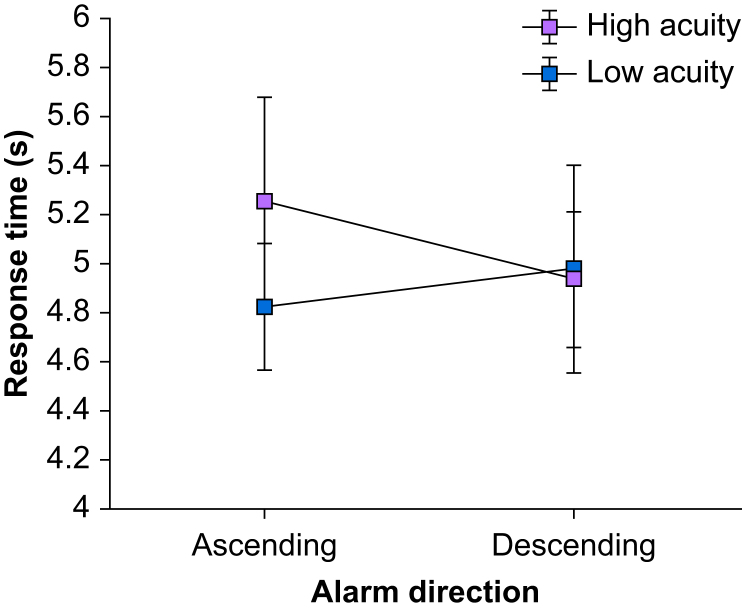
Interaction of alarm direction (ascending versus descending) and acuity (low *vs* high) on response time.

For the response time data, we identified participants who had experienced at least one presentation of each of the four alarm types in both blocks and had made at least one correct response to each of the four alarm types. Of the participants, 22 of 26 clinicians (85%) and 16 of 19 non-clinicians (84%) met these inclusion criteria for analysis. Using these participants, we ran a mixed model anova with response time as the dependent variable, pointer direction, acuity, and pointer classification (conventional *vs* improved) as within-subjects factors and participant type as a between-subjects factor. As in the prior response time analysis, we found a large main effect of participant type (*F*(1,36)=8.18, *P*=0.007, *η*^*2*^_*p*_=0.19). Non-clinicians (mean response time=4.60 s, 95% CI 4.18–5.03 s) made faster correct responses than did clinicians (mean response time=5.40 s, 95% CI 5.03–5.76 s). Furthermore, we found evidence for a medium-sized interaction between direction and acuity (*F*(1,36)=4.33, *P*<0.05, *η*^*2*^_*p*_=0.11). Figure 5 illustrates the interaction of pointer direction and acuity on response time. The right side of the figure shows that descending alarms did not differ in the time it took participants to respond to them, regardless of the alarms' acuity. However, as the left side of the figure shows, participants were slower in responding to low acuity, ascending alarms than they were at responding to low acuity, descending alarms. Furthermore, the mean for high acuity, ascending alarms (shown by the black square on the left side of the figure) did not differ significantly from either of the means for descending alarms (shown by the squares on the right side of the figure). In other words, ascending, low acuity alarms produced slower response times than the other combinations, which did not differ from each other.

## Discussion

There are advantages and disadvantages to different alarm design concepts. Earcons (abstract sounds with no ecological relationship to their referent) have proved inferior in terms of accuracy and response time,^20^ and with respect to subjective ratings by users. They often require user training and are less intuitive compared with other forms of alerting. Voice-based alerts are clear and highly specific and have resulted in better performance than auditory icons.^20^ However, they are less reliable in acoustically complex environments, as they take longer to process and can cause cognitive interference.^21^ Spearcons (accelerated or compressed speech) are faster and more compact than voice alerts but require significant cognitive effort to interpret when paired with multitasking.^22^ Auditory icons are natural, intuitive sounds that are highly effective for quick recognition, but may be less compatible with complex, abstract information and lack the specificity and directionality provided by pointers.^23^ Compared with voice alerts, pointers avoid overloading auditory channels and do not interfere with verbal working memory, though they may be less effective in highly dynamic environments where users cannot continuously monitor visual displays.^23^ For these reasons and for the purposes of our laboratory-based paradigm under controlled conditions, we found altering the pointer to be the most practical form of alarm design, as this was a simpler and more specific approach than evolving the auditory icon itself.

Three key points emerge from this study. The first is that our newest pointer significantly improves performance beyond what we have shown in our 2023 study.^10^ The second is that non-clinicians responded more quickly than clinicians, a finding which looks superficially anomalous. Lastly, differences in performance depend upon the signal's directionality, an asymmetry found in other auditory design and perception work.

Our primary finding is that enhancement of the ‘pointer’ associated with IEC 60601-1-8 alarms improved performance for both clinicians and non-clinicians in terms of task accuracy, without affecting response times. The pointers developed in Bingham and colleagues^10^ allowed for discrimination not only between the acuity of the two conditions (which is to some extent already encapsulated in the use of three-*vs* five-tone pointers in the standard) but also allowed for discrimination between upward and downward movement in blood pressure readings. The pointers devised for the current study are intended to indicate the same pieces of information but are acoustically enhanced.

Secondly, clinicians had slower response times than non-clinicians, likely because of their cautious decision-making approach influenced by professional obligations and situational awareness theory,^24^ the idea that our intrinsic goals and preconceptions influence our decision-making abilities. Conversely, non-clinicians, assumed to perceive lower risk and less pressure, responded faster. While response time differences between alarm types were not significant, the 0.69-s advantage with improved alarms could hold significance with respect to auditory processing happening on the order of 50–300 ms, though clinical relevance is still uncertain.

Lastly, our findings highlight a key challenge in auditory design. Although identical acoustic changes were used for both ascending and descending pointers, accuracy was higher with ascending cues. This suggests that upward changes are more perceptually salient than downward ones, even when the stimulus is the same. This bias aligns with prior research^18^ showing that upward changes in pitch, loudness, or tempo are perceived more intensely, likely because of evolutionary factors—approaching sounds signal greater threat than receding ones. Ascending tones are already widely used in alerts and warnings such as first responder sirens. The ideal alarm should be easily learned and retained with its associated clinical significance. Interestingly, subjective feedback did not suggest that descending stimuli were perceived as quieter.^25^^,^^26^ However, the reduced accuracy for downward changes points to a perceptual discrepancy that must be addressed in design.^27^, ^28^, ^29^, ^30^ To counter this, downward changes could be made more prominent, or additional cues could be added to reinforce downward movements.

Our controlled, laboratory-based paradigm does not reflect the complexity of multitasking and multisensory integration in true clinical settings, which presents a limitation in our study, in addition to this study being a single-centre study. Likewise, it is inherently challenging to simulate various environmental cues simultaneously and in a standardised manner, and to quantify the cognitive load of alarm systems outside of this experimental setting. However, for these same reasons, a laboratory-based approach presents an opportunity to observe and quantify the effects of our design in a highly calibrated and structured environment at low cost, strong reproducibility, and minimal to no ethical concerns. Preclinical experimental simulations informed by fieldwork studies can lay the groundwork alarm improvement and thus, risk management in clinical settings.

In this study, we have appealed to melodic intuition by delineating a specific set of associations distinguished by directionality, acuity, and harmony—concepts that humans are inherently familiar with in practice, regardless of our level of training. Future studies should focus on greater optimisation of auditory icons—sounds that convey information about an object, event, or situation. As mentioned in the discussion, our bias towards upward changes is an interesting phenomenon and has important implications for design. The idea of upward changes being perceived with better salience by the human ear may be a good steppingstone for future studies using pointers. Multisensory alarm systems (i.e. incorporation of visual cues) are also promising, as they could better reflect the true workflow and urgency of a clinical environment.

### Conclusions

Our improved pointer increases response accuracy by 10% in clinicians and non-clinicians without compromising response time. Adding harmonic complexity to cardiovascular auditory icons enhances efficacy and leverages human responses to directionality, roughness, and tone. Aligned with IEC standards, this design demonstrates compelling potential for improving clinician alarm identification accuracy and thus, advancing the landscape of technology-informed medicine in improving patient safety.

## Authors’ contributions

Data analysis, manuscript writing of all drafts, corresponding author for submission: MS

Final approval: MS, AB, JE

Study design: IG

Participant recruitment and administration of experimental trials: IG, RM, AL

Contributed to writing of first draft of the manuscript: RM, AL

Substantial contribution to auditory alarm design: TL

Contributed to writing of third draft of the manuscript: AB

Writing the thirds draft of the manuscript: JE

Lead data interpretation and analysis of results: JS

Study conception and experimental design, auditory alarm design, supervision of research, analysis, and manuscript: JJS

All authors listed above meet all four conditions to comply with ICMJE recommendations.

## Funding

Office of Naval Research (N00014-22-1-2184).

## Declarations of interest

The authors declare that they have no conflicts of interest.

## References

[1] Vockley M. (2015). https://www.aami.org/docs/default-source/foundation/alarms/alarm-compendium-2015.pdf.

[2] Cvach M. (2012). Monitor alarm fatigue: an integrative review. Biom Instrum Technol.

[3] Cvach M.M. (2014). Managing hospital alarms. Nurs Crit Care.

[4] Wee A.N., Sanderson P.M. (2008). Are melodic medical equipment alarms easily learned?. Anesth Analg.

[5] Arrabito G.R. (2009). Effects of talker sex and voice style of verbal cockpit warnings on performance. Hum Factor..

[6] Roche T.R., Braun J., Ganter M.T. (2021). Voice alerting as a medical alarm modality for next-generation patient monitoring: a randomised international multicentre trial. Br J Anaesth.

[7] Deschamps Ml, Sanderson P, Waxenegger H, Mohamed I, Loeb Rg (2024). Auditory sequences presented with Spearcons support better multiple patient monitoring than single-patient alarms: a preclinical simulation. Hum Factor..

[8] McNeer R.R., Horn D.B., Bennett C.L., Edworthy J.R., Dudaryk R. (2018). Auditory icon alarms are more accurately and quickly identified than current standard melodic alarms in a simulated clinical setting. Anesthesiology.

[9] IEC 60601-1-8:2006 (2006). https://www.iso.org/standard/41986.html#amendment.

[10] Bingham M.A., Cummins M.L., Tong A. (2023). Effects of altering harmonic structure on the recognition of simulated auditory arterial pressure alarms. Br J Anaesth.

[11] King R. (2010, August 10). https://web.archive.org/web/20100917053436/http://aami.org/news/2010/081010.press.606011.html.

[12] Saleem J.J., Russ A.L., Sanderson P., Johnson T.R., Zhang J., Sittig D.F. (2009). Current challenges and opportunities for better integration of human factors research with development of Clinical Information Systems. Yearb Med Inform.

[13] Table H. (2020). 1 of Medical Electrical Equipment—part 1-8: general requirements for basic safety and essential performance—collateral standard: general requirements, tests and guidance for alarm systems in medical electrical equipment and Medical Electrical Systems, Amendment 2. Int Electrotechnical Comm.

[14] Edworthy J.R., McNeer R.R., Bennett C.L. (2018). Getting better hospital alarm sounds into a global standard. Ergon Des.

[15] Edworthy J., Reid S., McDougall S. (2017). The recognizability and localizability of auditory alarms: setting global medical device standards. Hum Factor..

[16] Bruder A.L., Rothwell C.D., Fuhr L.I., Shotwell M.S., Edworthy J.R., Schlesinger J.J. (2021). The influence of audible alarm loudness and type on clinical multitasking. J Med Syst.

[17] Gadrey S.M., Clay R., Zimmet A.N. (2020). The relationship between acuity of organ failure and predictive validity of sepsis-3 criteria. Crit Care Explor.

[18] Deutsch D., Hamaoui K., Henthorn T. (2007). The glissando illusion and handedness. Neuropsychologia.

[19] Schutz M., Stefanucci J.K., Baum S.H., Roth A. (2017). Name that percussive tune: associative memory and amplitude envelope. Q J Exp Psychol (Hove).

[20] Nees M.A., Liebman E. (2022).

[21] Neary A., Li S.Y.W., Salisbury I., Loeb R.G., Sanderson P.M. (2023). Effects of multitasking on interpreting a spearcon sequence display for monitoring multiple patients. Appl Ergon.

[22] Davidson T., Ryu Y.J., Brecknell B., Loeb R., Sanderson P. (2019). The impact of concurrent linguistic tasks on participants’ identification of Spearcons. Appl Ergon.

[23] Lei Z., Ma S., Li H., Yang Z. (2022). The impact of different types of auditory warnings on working memory. Front Psychol.

[24] Endsley M.R. (2015). Final reflections. J Cogn Eng Decis Mak.

[25] Neuhoff J.G., Wayand J., Kramer G. (2002). Pitch and loudness interact in auditory displays: can the data get lost in the map?. J Exp Psychol Appl.

[26] Semal C., Demany L. (2006). Individual differences in the sensitivity to pitch direction. J Acoust Soc Am.

[27] Edworthy J., Hellier E., Aldrich K., Loxley S. (2004). Designing trend-monitoring sounds for helicopters: methodological issues and an application. J Exp Psychol Appl.

[28] Neuhoff J.G. (1998). Perceptual bias for rising tones. Nature.

[29] Neuhoff J.G. (2019). Proceedings of the 25th International Conference on Auditory Display (ICAD 2019).

[30] Vos J.J. (1978). Colorimetric and photometric properties of a 2° fundamental observer. Color Res Appl.

