# Supplementary material for: Improved pointer in auditory alarms enhances response accuracy
Source: BJA Open. 2025 Mar 13;14:100379. doi: 10.1016/j.bjao.2025.100379 (PMC11952019; doi:10.1016/j.bjao.2025.100379)
Supplement: Multimedia component 1 [file mmc1.docx]

**Design of the Improved Pointer**

In the study reported here, we take the auditory pointer developed specifically for cardiovascular use previously utilized in Bingham et al, and evolve it with harmonic complexity, roughness (the harsh, raspy nature of narrow harmonic intervals best correlated with sensory dissonance) and glissando (auditory gliding that enables smooth flow from one note to another (typically of different pitch) to create distorted sounds between the starting and ending notes to differentiate between low and high acuity. Our goal was to supplement the pointer with more information for the end user and make this auditory signal perceptually intuitive (Deutsch et al 2007; Schutz et al, 2017). We chose these design elements based on the basic capabilities of non-musicians to perceive and derive meaning from sound. Since we have demonstrated absolute improvement in speech perception and visual vigilance with use of the auditory icon in clinicians (Bruder et al 2021), we now expand our non-musician cohort to include those without medical background - both clinicians and non-clinicians. Further, given this is a stepwise evolution of design, we hypothesized improved accuracy and non-significant improvement in response time, and that non-clinicians would not perform appreciably worse than clinicians due to intuitive design. We incorporated direction of pitch change (ascending vs descending) to better orient the user to change. With varying intervallic differences (by addition of 3, 5, and 7 odd harmonics to the final note), we discriminate between moderate and severe acuity. We incorporated consonant-to-dissonant perceptual change which has been proven by our colleagues to improve alarm identification (Bingham et al, 2023). Our objective is to offer a perceptual training paradigm that is still somewhat novel to anesthesiology, but that has been used successfully in basic science research settings in the field of sensory perception as well as in a growing body of evidence with similar. We believe that our findings are generalizable to anesthesia training programs nationwide. While future work should seek to extend these results to more realistic clinical settings, these findings are a first step in a line of research that has the capacity to save lives and minimize errors in the operating room by using effective and time-efficient multisensory training to improve an anesthesia provider’s subtle unisensory pulse oximetry pitch perception in a dynamic and attentionally demanding perioperative environment. Clinicians often face the challenge of dividing attention and prioritizing tasks, especially when their cognitive resources reach their maximum capacity. Research published in anesthesiology literature shows that using multisensory cues, combining visual and auditory inputs, can improve perception, accuracy, and response times, enhancing overall performance. In this study, we quantify performance by measure of auditory response time and accuracy. Rather than hypothesizing that our experimental design will result in clinical significance, again, we hypothesize improvement from previous alarm designs, and hope to add to the body of applied studies that demonstrates the psychological, neuroscience, and human factors aspects of improving clinicians’ abilities via alarm optimization.
